# Biomimetic Digital Twin of Future Embodied Internet for Advancing Autonomous Vehicles and Robots

**DOI:** 10.3390/biomimetics10110774

**Published:** 2025-11-14

**Authors:** Ming Xie, Xiaohui Wang

**Affiliations:** 1 School of Mechanical and Aerospace Engineering, Nanyang Technological University, 50 Nanyang Avenue, Singapore 639798, Singapore; mmxie@ntu.edu.sg; 2 Shenyang Institute of Computing Technology, Chinese Academy of Sciences, Shenyang 110168, China; 3 University of Chinese Academy of Sciences, Beijing 100049, China; 4 Liaoning Key Laboratory of Domestic Industrial Control Platform Technology on Basic Hardware and Software, Shenyang 110168, China

**Keywords:** digital twin, inter-process communication, RobotX Challenge, robotics operating system, autonomous systems, software port

## Abstract

Efficient coordination among software modules is essential for biomimetic robotic systems, much like the interaction among organs in a biological organism. However, implementing inter-process or inter-module communication in autonomous systems remains a complex and time-consuming task, particularly for new researchers. Simplifying inter-module communication is the central focus of this study. To address this challenge, we propose the DigitalTwinPort framework, a novel communication abstraction inspired by the port-based connectivity of embedded hardware systems. Unlike middleware-dependent solutions such as ROS, the proposed framework provides a lightweight, object-oriented structure that enables unified and scalable communication between software modules and networked devices. The concept is implemented in C++ and validated through an autonomous surface vehicle (ASV) developed for the RobotX Challenge. Results demonstrate that the DigitalTwinPort simplifies the development of distributed systems, reduces configuration overhead, and enhances synchronization between digital and physical components. This work lays the foundation for future digital twin architectures in embodied Internet systems, where software and hardware can interact seamlessly through standardized digital ports.

## 1. Introduction

The pursuit of autonomous systems that emulate the resilience and adaptability of biological intelligence has created a pressing need for robust frameworks capable of mirroring and predicting the state of physical entities in dynamic environments. This challenge is particularly evident in maritime robotics, as exemplified by competitions such as the RobotX Maritime Challenge [1]. In these contexts, the concept of a Digital Twin has emerged as a transformative paradigm for managing complex, bio-inspired systems [2]. By creating a bridge between the physical and digital worlds, Digital Twins enable enhanced analytics, performance monitoring, and predictive maintenance—key enablers for advancing autonomous systems operating within the expanding Internet of Things (IoT) ecosystem [3].

As we progress towards the “Future Embodied Internet”, the integration of Digital Twins with autonomous technologies becomes crucial [4]. This integration is envisaged not just to streamline the operations of autonomous vehicles and robots but also to ensure their safe and efficient functioning within an interconnected framework. In this paper, we delve into the concept of a Digital Twin dedicated to the future embodied internet, focusing on its application in environments like those presented in the RobotX Maritime Challenge, where it depicts that autonomous vehicles and robots often face unique and dynamic conditions [5,6].

The application of Digital Twins [7,8] in such domains is fraught with challenges. The dynamic nature of autonomous systems, characterized by their need for real-time data exchange, integration of complex modules (which are needed for Sensing, Perception, Planning and Control), and adaptive control mechanisms, requires an equally dynamic and resilient virtual counterpart. Moreover, the vast volume of data and the imperative of timely and accurate data interpretation add to the complexity. This paper identifies these challenges and sets forth a problem statement that guides the subsequent discourse.

In charting the course for this research, we explore the existing landscape through a literature review, identify the current limitations of prevalent platforms like ROS (Robot Operating System) and Simulink in constructing Digital Twins, and propose a forward-looking development framework. Central to this framework is the proposed ‘port’ class in C++, which is relevant to any Object-Oriented Programming Language, designed to act as a cornerstone in the communication between networked software modules—such as camera, hydrophone, GPS modules—and the networked devices they control. This class aims to simplify connectivity and enhance the modularity of the Digital Twin’s architecture, thereby offering a significant contribution to the field.

**Figure 1:**
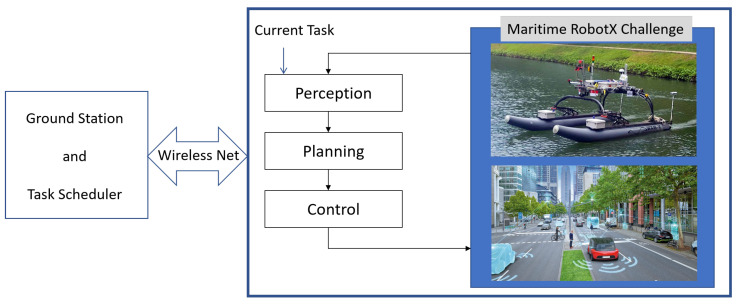
Research Framework Underlying the Development of Autonomous Surface Vehicles.

As depicted in Figure 1, each task requires perception, planning, and control to execute, which involves several sensors that are connected to several different physical ports. The interface of the physical ports and software modules is often not straightforward and confusing. Therefore, this paper presents a new way to configure and connect the software modules in developing communication networks for autonomous vehicles and robots.

This paper is organized as follows: In Section 2, we will outline the problem statement. In Section 3, we will survey the current, existing, and common solutions. In Section 4, we will present the study case in ROS in RobotX Maritime Challenge and Simulink. In Section 5, we will present the limitations of ROS and Simulink. Section 6 presents the details of the proposed solution and the steps to implement it. Lastly, the conclusion and some future works/considerations are detailed in Section 7.

## 2. Problem Statement

**Figure 2:**
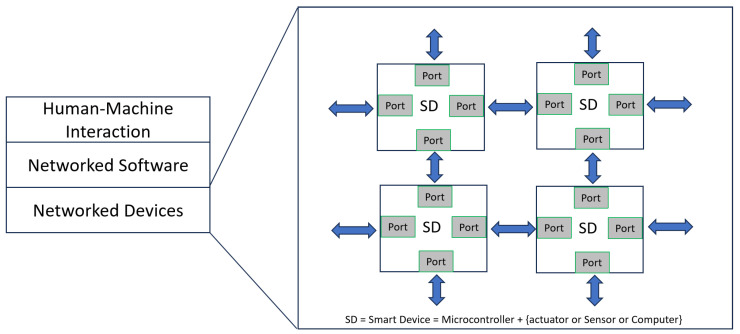
Communication Architecture among Intelligent Devices in the Autonomous System.

The ever-increasing complexity of autonomous systems has precipitated a critical need for sophisticated interconnectivity between disparate software modules to facilitate seamless data exchange. Figure 2 illustrates the quintessential architecture of such a system, highlighting the integration between Human-Machine Interaction, Networked Software, and Networked Devices, all interconnected through multiple ‘Ports’. The ‘Smart Device’ (SD) nodes—each potentially a microcontroller combined with an actuator, sensor, or computer—require a framework that can handle the bi-directional flow of information reliably and efficiently.

The necessity for inter-module communication is underscored by scenarios such as coordinated navigation, where multiple autonomous vehicles, each equipped with their Digital Twin, must share sensory data, environmental analyses, and operational statuses to navigate and operate collaboratively in real-time. In such scenarios, a failure in the communication fabric can lead to critical errors, mission failure, or even catastrophic system breakdowns. As these systems scale, the volume of data exchange increases exponentially, presenting significant challenges in maintaining system integrity and performance.

This paper identifies the gap in a standardized approach to interconnect software modules within autonomous systems’ Digital Twins. The integration complexity not only hinders the system’s scalability and adaptability but also restricts the potential for collaborative development and innovation. Hence, there is a pronounced need for a universal, open standard ‘port’ class that can be effortlessly implemented across different platforms and programming paradigms, facilitating robust and secure data exchange and system interoperability.

## 3. Related Works

**Figure 3:**
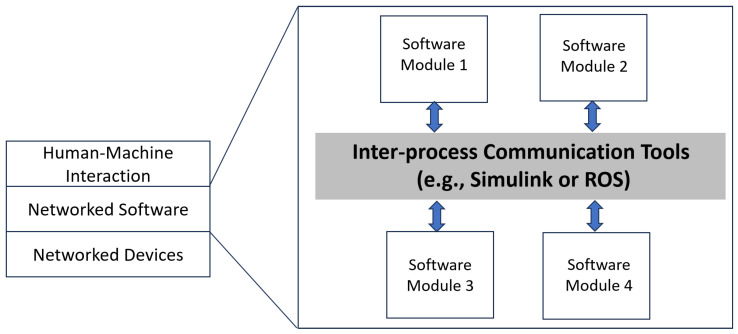
Internal Connections within Autonomous Systems Autonomous of Robot.

The orchestration of inter-process communication (IPC) within the domain of autonomous systems [9] has been a cornerstone of research and development, particularly in the context of Digital Twins. This segment of the paper, illustrated by Figure 3, examines the current state of IPC tools, with a specific focus on two of the most prevalent platforms in this space: Robot Operating System (ROS) and Simulink.

### 3.1. Use of ROS to Achieve Inter-Module Communication

ROS, on the other hand, is an open-source middleware suite that has become synonymous with robotics development. Its modular design and publisher-subscriber communication model enable a distributed processing architecture, which is essential for the complex sensor and actuator networks typical in robotics [9].

### 3.2. Use of Simulink to Achieve Inter-Module Communication

Simulink, a proprietary tool developed by MathWorks, has been widely adopted for model-based design and multi-domain simulation. Its graphical programming environment allows for simulation and automatic generation of code, which is crucial for testing and deploying control systems. Studies have shown Simulink’s effectiveness in designing complex systems, such as autonomous vehicle algorithms, which require rigorous testing across various simulated environments before physical implementation [10,11].

### 3.3. ROS and Simulink as Digital Twins

Despite their widespread use and robust capabilities, both Simulink and ROS have recognized limitations. Simulink’s closed-source nature and cost can be prohibitive, while ROS’s lack of real-time guarantees and steep learning curve present significant barriers to entry [12,13,14]. Researchers and practitioners have highlighted these issues, noting that while powerful, these tools require considerable expertise and resources to utilize effectively.

The need for standardized IPC tools that can provide the flexibility of ROS with the model-based design capabilities of Simulink is evident. Such tools would not only facilitate easier integration of software modules across different platforms but also promote a more inclusive environment where developers can contribute to the advancement of autonomous systems without the overhead of mastering complex, platform-specific details.

The integration of Digital Twins with IPC tools like ROS and Simulink is depicted in Figure 3. The figure delineates the flow of information between software modules and emphasizes the central role of IPC in maintaining the fidelity of Digital Twins. As the field progresses, there is a growing consensus on the need for IPC mechanisms that can accommodate the evolving demands of Digital Twins, particularly in terms of standardization, real-time processing, and ease of use [15].

## 4. A Case Study of Using ROS to Implement Digital Twin of Internet for Autonomous Surface Vehicles

### 4.1. Description of a Specific Task in RobotX 2022

The RobotX Challenge 2022 involves several tasks, and each task requires a combination of various sensor functionalities. To give a better understanding of the task, one of the tasks is described below.

**Figure 4:**
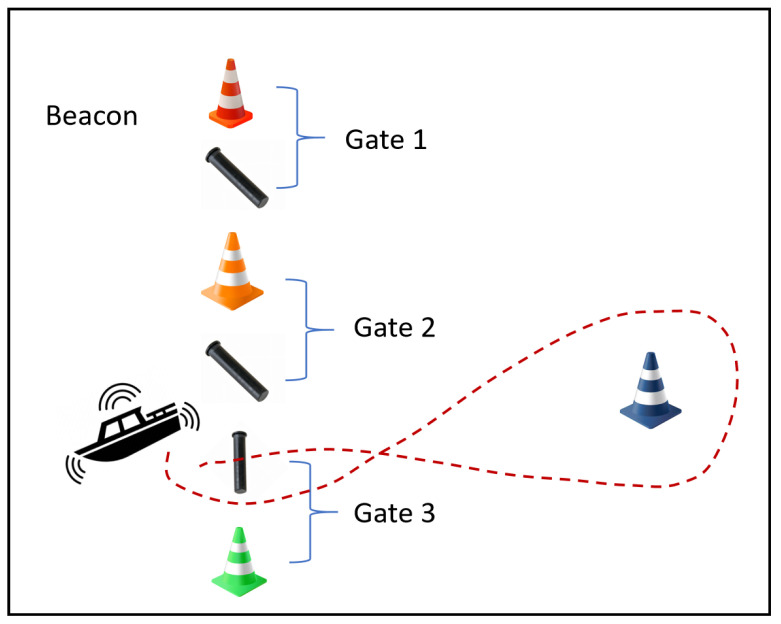
Task 2 scenario in RobotX 2022 showing gate navigation sequence and buoy circling requirement for Autonomous Surface Vehicles. The dotted line indicates the navigation path.

Task 2 requires an autonomous surface vehicle (ASV) [16,17] to adeptly enter and navigate through a sequence of gates, responding to an active beacon emitting a known frequency. The task culminates in the ASV encircling a designated floating object, in this instance represented by a black buoy. Figure 4 illustrates the trajectory and the strategic placement of gates and buoys, providing a visual reference for the task’s complexity. Critical to the ASV’s success is the integration of various sensors—cameras, hydrophones, and GPS—which collectively provide the vehicle with situational awareness and positional accuracy. These sensors enable the ASV to chart a collision-free path through the gates, while also maintaining proximity to the beacon and the target buoy.

### 4.2. Task 2 of RobotX 2022 Configuration in ROS

**Figure 5:**
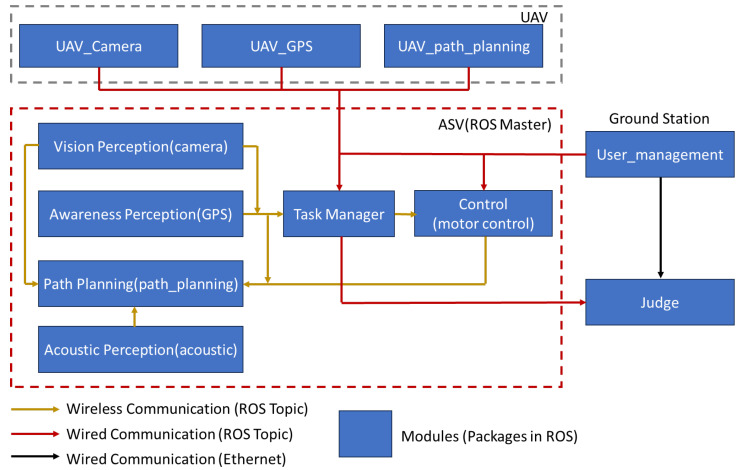
ROS package-level architecture for Task 2, depicting modular decomposition into vision perception, GPS localization, path planning, and acoustic perception subsystems.

In order to configure this task in ROS, we have to first configure the ROS packages level. The system’s architecture is designed to be modular, with distinct ROS packages such as UAV_camera, UAV_GPS, and UAV_path_planning dedicated to specific functionalities. These packages correspond to the critical sensor systems required for the ASV to navigate the course successfully. The Vision Perception package processes visual data from the camera, the Awareness Perception package handles GPS data for location tracking, and the Path Planning package manages the navigation strategy. An additional package, Acoustic Perception, is incorporated to process signals from the hydrophone, detecting the beacon’s frequency for gate identification. Please refer to Figure 5 for a better understanding of the ROS packages configurations.

Specific to task 2 in Maritime RobotX Challenge 2022, Figure 6 shows the communication among the nodes. Leaving aside the details if using a Navigation Stack packages provided by ROS, such as move_base which will require a lot more topics to be configured. In the following section, the description of each node and topics will be covered respectively.

There are a total of 7 nodes (excluding Judge) and 7 topics. They are explained further below.

Task 2 Nodes

1. task_manager/task_checker: This node acts as a switchboard, determining whether the conditions are met to proceed with Task 2. It continuously listens for updates from the overarching task management system to ensure synchronization with the mission’s current phase.
2. task_manager/task2: It publishes status updates to topics that other nodes are subscribed to, such as user_management/task_status to update the GUI.
3. path_planning/path_task2: After receiving a trigger from the task_checker, this node initiates the navigation process. It calculates the vehicle’s trajectory to navigate through the designated gates. The computed path accounts for environmental data and vehicle dynamics to optimize the route for safety and efficiency.
4. gps/ASV_gps: This node provides geographical data that is essential for path planning. By furnishing precise location coordinates, it ensures the vehicle stays on course and corrects the trajectory in response to any positional drifts or external disturbances.
5. camera/cam_task2: Serving the dual purpose of obstacle detection and visual feedback, this node processes the imagery data for real-time decision-making. It assists in identifying the gates and any intervening obstacles, facilitating autonomous navigation and ensuring adherence to the competition’s rules.
6. acoustic/beacon_detector: Specialized for acoustic signal processing, this node listens for and interprets underwater acoustic beacons that may be part of Task 2’s requirements. The detection of these signals can influence the vehicle’s path, especially if the task involves localizing acoustic sources.
7. user_management/GUI: As the interface between the system and the user, this node presents real-time information on the task’s status. It also allows for manual overrides or adjustments, ensuring that the operator can intervene if necessary.

Task 2 Topics

1. /task_status: This topic carries messages indicating the current status of Task 2. The message is used to communicate the specific stage of the task, which in this case is the Entrance and Exit Gates task.
2. /task2/ASV_gps: The ASV GPS topic provides geographic positioning data crucial for navigation. The messages shown include coordinates along with a timestamp and an 30 identifier for the robot, ensuring that navigation is based on the most recent and accurate location information.
3. /task2/beacon_freq: This topic is used to publish the frequency of an acoustic beacon detected by the system. This information is typically used to make navigation decisions when acoustic beacons are a part of the task, helping the ASV to locate objects or navigate to specific points.
4. /task2/cam_task2: The camera node for Task 2 outputs data related to visual elements detected by the camera system. The message indicates the detected position of an object, presumably a gate in this context, within the camera’s frame of reference.
5. /task2/entrance_exit_gate: This topic conveys the status of entrance and exit gates, likely as part of a challenge where the ASV must pass through gates in a specific order. The data includes an identifier for the robot, the status of gates, and a timestamp.
6. /task2/propeller_speed: This topic informs about the command velocity and orientation for the propellers of the ASV. The message commands the speed and direction of the ASV’s movement, critical for maneuvering through the course of Task 2.
7. /task2/which_gate: This topic specifically carries messages regarding the gates the autonomous surface vehicle (ASV) must navigate through during Task 2. The data clearly indicates which gates are designated as the entrance and exit for this phase of the task.

**Figure 7:**
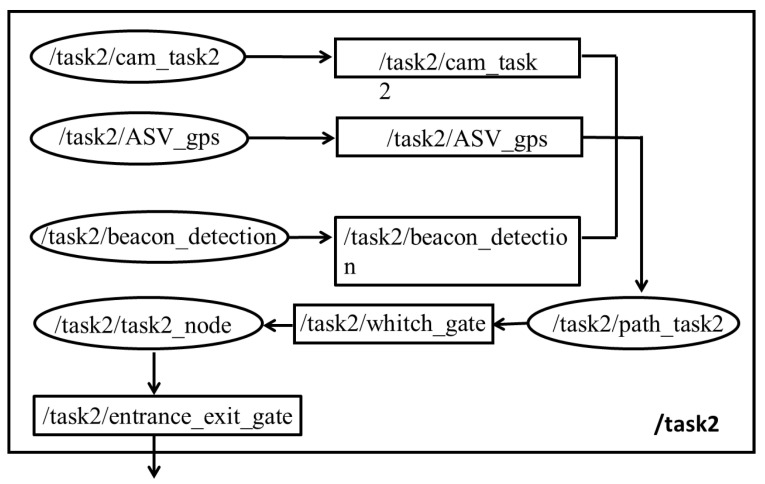
Clean rqt_graph visualization of Task 2 with namespace organization (‘/task2’), demonstrating improved clarity through logical grouping of nodes and topics.

**Figure 8:**
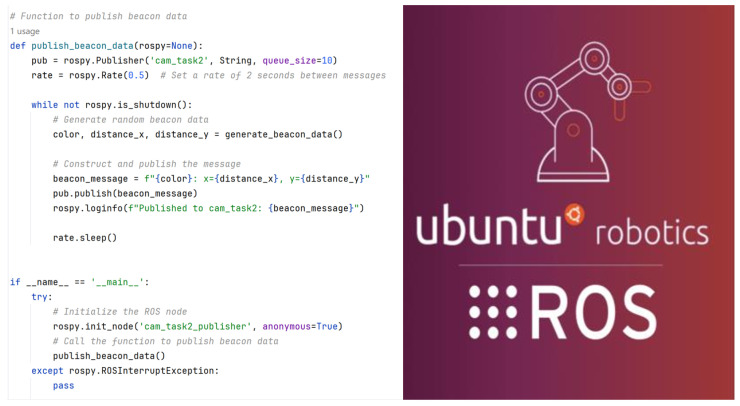
The Configuration code for Task 2.

Additionally, ROS provides a tool to visualize the running nodes as well as the topics associated with it, this tool is called rqt_graph. This configuration could be quite hard and confusing to visualize. When the user requested the rqt_graph of the running task, it could be quite messy as one node may publish several topics. This visualization could be further improved if the user provides the namespace when configuring the node. This namespace will now group the same nodes that come from the same namespace. Figure 7 shows the configuration of task 2 with namespace ‘/task2’, and Figure 8 shows the configuration code of task 2.

The rqt_graph visualization in Figure 7 demonstrates how namespace organization (‘/task2’) significantly improves graph clarity by logically grouping related components, addressing the complexity of multi-topic node communication.

## 5. Limitations of ROS and Simulink

### 5.1. Limitations of ROS

The application of ROS has been instrumental in advancing the field of robotics, providing a framework for writing software for robotic systems [18,19]. However, when it comes to the specific application of ROS in the implementation of Digital Twins for autonomous systems, several limitations become apparent.

In Section 4.2, it is shown how the communication in ROS is typically configured. It can be seen that it is quite tedious task to set everything up. Keep in mind that this is only configured for 1 task, and there are a total of 8 tasks in RobotX Challenge 2022, and each need to be configured individually by keeping track what package and node to be used. Some major limitations will be further elaborated below.

1. Complexity of Setup and Inappropriateness of Terminologies: While ROS offers flexibility and a rich set of functionalities, setting up a ROS environment can be complex and time-consuming [20]. The configuration of nodes, topics, and services necessitates a steep learning curve. This complexity can be a barrier, especially for unaccustomed users who may not have extensive experience with such middleware systems. Additionally, the terminology used in ROS may cause confusion for the first time users. The term of publisher, subscriber, and topics are not standardized for engineer and is more directed towards software developer who normally comes from Computer Science background.
2. Scalability Issues: As the number of interconnected devices and the volume of data grow, ROS may struggle to scale efficiently. Its network-based communication can become a bottleneck in large-scale systems, where numerous messages are being passed simultaneously, potentially leading to increased latency and reduced system performance.
3. Limited Support for Non-Linux Platforms (not a platform independent): ROS has traditionally been tied closely to Linux [21], and while there have been efforts to port ROS to other operating systems, support remains limited. This restricts the environments in which a ROS-based Digital Twin can be developed and deployed. Similarly to security concern, ROS 2 may have address the issues but some configurations have to be done.

### 5.2. Limitations of Simulink

Simulink on the other hand is the block diagram environment based on MATLAB R2022b may be beneficial for small scale robotic systems. Some limitations of Simulink will be further explored below.

1. Performance: Simulink models may not be as efficient as hand-written code, especially for high-performance or real-time applications. The overhead of the simulation environment and the abstraction layers can introduce latency and slow down execution.
2. Flexibility and Control: In conventional coding, programmers have fine-grained control over every aspect of the program, which can be critical for optimizing algorithms and handling specific hardware or system requirements. Simulink’s abstraction might limit this detailed control.
3. Complexity and Scalability: For very complex systems, a Simulink model can become large and unwieldy, making it difficult to manage and understand. In contrast, conventional code can be organized into modules, libraries, and services that can be more easily scaled and maintained.
4. Cost and Accessibility: Simulink requires a license, which can be quite costly, whereas many programming languages and development environments are open source and free to use.
5. Customization: While Simulink provides many built-in blocks and functions, creating custom functionality can be more complex compared to writing code directly in a programming language where you can define exactly what you need.
6. Version Control: Managing versions and changes in Simulink models is not as straightforward as with text-based code, which fits seamlessly into version control systems like Git.
7. Execution Environment: Simulink models require MATLAB to run, whereas conventional code can often be compiled into standalone applications or run in lightweight environments.
8. Portability: Models created in Simulink are inherently less portable than conventional code because they rely on the MATLAB/Simulink environment. Conventional code can be compiled and run on any machine with the appropriate compiler and runtime.

## 6. Proposed Future Development Toward Digital Twin of Internet

### 6.1. Implementation of the DigitalTwinPort Framework

**Figure 9:**
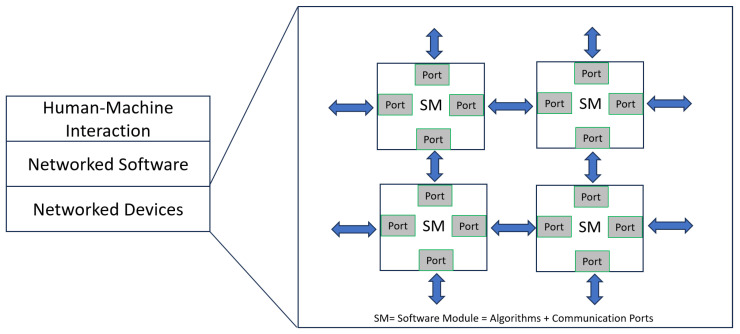
Internal Parallel Relationships in Communication between Software Modules within Autonomous Systems.

In Figure 9, the structural parallel between the ‘Networked Software’ layer and the ‘Networked Devices’ layer encapsulates a key principle of digital twin architecture—the reflection of physical devices in the digital domain through software modules.

Each ‘Software Module’ (SM) within the Networked Software layer corresponds to the ‘Smart Device’ (SD) in the Networked Devices layer. They communicate through digital twin ports that emulate physical interfaces, supporting bidirectional data exchange and synchronization between virtual and physical systems. The proposed digital twin communication layer enables unified and scalable communication between software modules and networked devices. This architecture not only supports individual ASV control but also scales naturally to multi-ASV collaboration, where multiple DigitalTwinPort instances can interconnect to form a distributed communication network. This correspondence is not merely representational but functional. The SMs contain algorithms that process data, akin to how a device processes inputs and outputs. They also feature digital twin communication ports that enable them to send and receive data, comparable to the physical ports on networked devices.

The diagram suggests that each physical device in the network is paired with a dedicated software module. The ports surrounding each SM indicate multiple points of interaction, which can represent different channels or types of data (e.g., sensor data, control signals, status updates) that the SM can handle.

Just as networked devices might be connected to various sensors, actuators, and other devices, the software modules are interconnected, forming a complex web that allows for the integration of data streams and control mechanisms. This interconnectivity within the digital layer is essential for managing and coordinating the diverse and often concurrent processes occurring within the physical layer.

The DigitalTwinPort class is proposed here, as part of this paradigm, offers a modular and flexible approach to networking that could significantly streamline the development of such complex systems.

**Figure 10:**
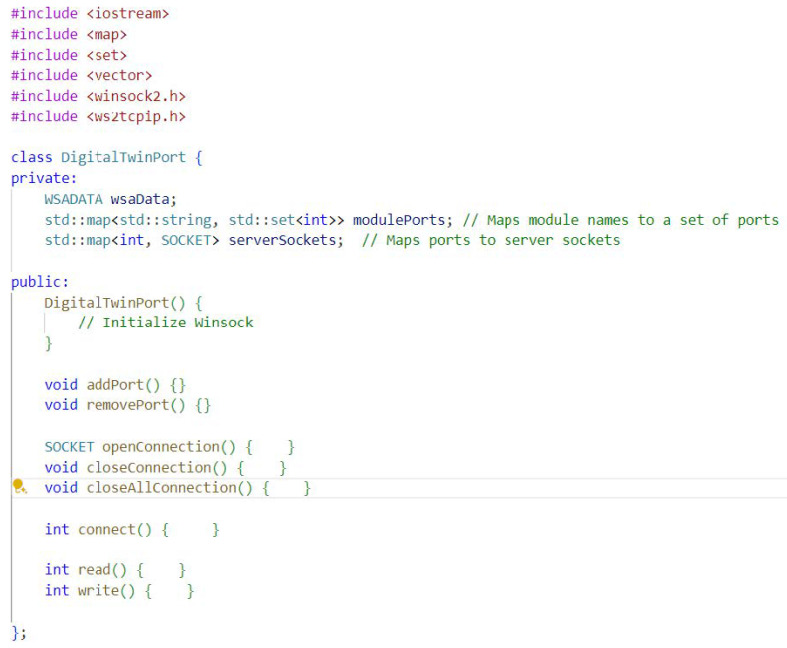
The proposed method code framework.

The proposed DigitalTwinPort class simplifies the process of establishing networked communication between software modules and hardware devices by abstracting away low-level networking details. The code framework for the proposed method is shown in Figure 10, and the complete code structure can be found in Appendix A. Unlike ROS, which requires manual configuration of nodes and topics, and Simulink, which operates in a block-based structure, the DigitalTwinPort class automates socket creation, connection handling, and data transmission. This class offers a more flexible, modular solution compared to the rigid, block-based structure of Simulink, and its object-oriented design makes it more adaptable and scalable for complex applications in autonomous systems. In large-scale autonomous fleets or multi-robot systems, each module can instantiate its own DigitalTwinPort, enabling peer-to-peer communication and distributed coordination without centralized middleware dependencies. By streamlining communication and reducing setup complexity, the DigitalTwinPort class significantly enhances development efficiency and enables faster deployment of networked autonomous systems.

Beyond mimicking physical ports, the DigitalTwinPort class embodies biomimetic principles by facilitating real-time, dynamic communication between software modules and hardware devices. Just as biological systems, like the nervous system, allow different organs and sensors to communicate and adapt to changing conditions, the DigitalTwinPort ensures that software modules can adapt to and respond to environmental changes. Additionally, the use of multiple ports and communication pathways within the system ensures redundancy and resilience, similar to how biological systems maintain robustness through interconnected organs and processes.

Here’s how the DigitalTwinPort class simplifies network communication:

1. Abstraction of Winsock Initialization: The constructor DigitalTwinPort() encapsulates the initialization of Winsock, the Windows library required for network communications. This hides the setup details from the user, who no longer needs to write boilerplate code for Winsock startup procedures.
  Although the current prototype is implemented using Winsock on Windows, the DigitalTwinPort framework is platform-independent by design. Its socket operations can be directly mapped to POSIX sockets on Linux systems, enabling seamless integration into ROS or ROS2 environments as a node wrapper or bridge for inter-module communication.
2. Simplified Port Management: The addPort() method abstracts the process of creating a socket, binding it to a port, and setting it to listen for incoming connections. Users can add ports without understanding socket creation and binding details, which are typically required in socket programming. And vice versa, the removePort().
3. Seamless Connection Handling: With openConnection() and closeConnection(), establishing and terminating connections is simplified into single method calls. Users don’t need to worry about the underlying call sequence of socket, bind, listen, and accept functions to establish a server socket, nor the nuances of disconnecting and closing a socket. On the other hand, closeAllConnection() is to shut down all connections of that server.
  connect(), initiates a client connection to a server with the given IP address and port. This method abstracts the client-side socket connection process, making it easier for the digital twin to connect to other networked entities.
4. Data Transmission Simplification: The read() and write() methods provide easy-to-use interfaces for data transmission. These methods would internally manage the send and receive socket calls, including the handling of the buffers and ensuring that the complete message is sent or received.
5. Default Port Configuration: The initializeDefaultPorts() method sets up a predefined set of ports for each software module, eliminating the need for manual configuration. This is especially useful for setting up standard ports for common services or devices within a digital twin environment.
6. Overall, the DigitalTwinPort class is proposed as a high-level interface for managing network communications tailored to digital twin implementations. It streamlines development by providing a set of easy-to-use methods for network operations, which would otherwise require a solid understanding of the underlying network programming concepts. This allows developers to focus more on the logic of their digital twin applications rather than the details of network programming.

### 6.2. Digital Twin Requirements and Mapping to DigitalTwinPort

A true digital twin requires not only connectivity but also synchronization of system states, time management, event tracking, and versioning of states. While the DigitalTwinPort focuses on simplifying networked communication, it can complement and enable several digital twin-specific services. These include:

- State Synchronization: DigitalTwinPort mirrors the physical state of devices, such as the position, velocity, sensor readings, and actuator statuses, allowing software modules to stay synchronized with their physical counterparts. In the ASV scenario, the real-time state of the vessel is continuously mirrored by its digital twin, ensuring accurate feedback and control.
- Event Logging: DigitalTwinPort can log events, such as sensor activations or control command updates, which can be used for debugging, replay, and system performance analysis.
- Replay Mechanism: With event logging in place, DigitalTwinPort can allow for event replay, enabling engineers to recreate scenarios and test system responses to disturbances or faults. This is useful for validating the system’s robustness under various conditions.
- Versioning: DigitalTwinPort can be extended to support versioning of the state of each software module, allowing for easy comparison of different versions of the system over time.

These services ensure that DigitalTwinPort aligns with the key characteristics of digital twin architectures and enhances the fidelity of virtual-physical system integration.

### 6.3. Security, Safety, and Real-Time Behavior

The DigitalTwinPort framework incorporates essential security, safety, and real-time mechanisms through minimal code additions, providing fundamental protection for autonomous maritime systems.

**Security Framework**

The security implementation focuses on authentication and access control through a token-based system. Each module must validate its identity using pre-shared tokens, with critical ports requiring successful authentication before establishing connections. A basic challenge-response handshake protocol prevents unauthorized access to safety-critical systems. Message validation ensures basic integrity by enforcing size limits and rejecting malformed data, while mutex locks provide thread safety for concurrent operations.

**Real-Time Performance**

Real-time responsiveness is achieved through timeout mechanisms in all blocking operations. Connection attempts automatically abort after predefined intervals, and read operations include configurable timeout parameters to prevent system hangs. The framework provides execution time monitoring to track task completion against requirements, ensuring predictable system behavior under normal operating conditions.

**Maritime Safety Case**

**Case Study: Basic Failure Handling** The framework implements fundamental failure response mechanisms through centralized handling functions. When system failures are detected, the framework triggers appropriate safety responses based on failure type. For critical failures, the system executes predefined safety protocols while maintaining communication integrity through authenticated channels. This approach ensures that basic safety functions remain operational during system anomalies, providing a foundation for more sophisticated safety architectures.

The DigitalTwinPort approach demonstrates that core security and safety requirements can be addressed through focused, minimal implementations, establishing a baseline for autonomous system protection while maintaining the lightweight characteristics essential for embedded maritime applications.

### 6.4. Comparison with Existing Middleware Frameworks

To clarify the novelty and positioning of the proposed DigitalTwinPort, this subsection compares its conceptual and functional features with those of existing middleware frameworks, including ROS, DDS, and MQTT-based message buses. While these middleware systems have established robust paradigms for inter-process communication through publish/subscribe or request/response mechanisms, they often rely on centralized registries, predefined message schemas, and external configuration files. These dependencies increase system coupling and reduce modular flexibility, particularly in lightweight or rapidly prototyped autonomous systems.

The DigitalTwinPort differs from these middleware abstractions in both design philosophy and implementation strategy. It introduces a port-level communication abstraction that mirrors the behavior of hardware ports within embedded devices. Each software module encapsulates its own communication endpoints through dedicated DigitalTwinPorts, which locally manage data binding, naming, and transport initialization without requiring a global discovery mechanism or middleware-specific XML/IDL definitions. This approach supports self-contained module design, enabling flexible module reuse and reducing integration overhead.

Moreover, the DigitalTwinPort aligns with the biomimetic principle underlying this work: software modules behave as self-contained “organs,” while DigitalTwinPorts function as “nerves” connecting them within the digital twin body. This unified design bridges physical and digital connectivity, facilitating scalable and interpretable module interaction for embodied digital twins.

Table 1 summarizes the distinctions between the proposed framework and widely used middleware systems. The comparison highlights that DigitalTwinPort offers a lightweight, object-oriented structure for module-level communication, while maintaining compatibility with standard network transport protocols.

This comparison demonstrates that the proposed framework operates at a finer granularity than traditional middleware, focusing on module-level autonomy and internal communication abstraction. By integrating communication logic directly within the module’s object structure, DigitalTwinPort simplifies the realization of digital twin architectures for autonomous systems, reducing configuration complexity while maintaining interoperability and scalability.

## 7. Conclusions

In this paper, we have outlined the concept of digital twin of future embodied internet which leverages on the use of the concept of digital twin of port corresponding to any microcontroller’s physical port. By mimicking the physical network of devices, the networked software layer can run simulations, predict outcomes, and provide insights based on the data it receives. This enables a proactive approach to system management and decision-making, where potential issues can be identified and addressed in the digital realm before they manifest physically.

The central concept proposed here is the digital twin’s ability to replicate the network’s physical complexity in a virtual space, where it can be managed more flexibly and safely. This capability is fundamental to the digital twin’s value proposition, offering a robust scalable framework for the predictive and reactive control of complex, interrelated systems, and a much faster network configuration development. This will save engineers much time in configuring the network which will require a few hours to understand and implement down to few minutes to implement. Given that the current proposed DigitalTwinPort is to establish connections and to send and receive messages/data, the future development of the DigitalTwinPort class could include machine learning algorithms to analyze patterns within network traffic, predicting potential failures or bottlenecks before they occur. This would enhance the proactive capabilities of digital twins. Rigorous testing and implementation in real-world scenarios will also provide insights into the practical challenges and opportunities of deploying DigitalTwinPort in more various industries.

Future work will focus on validating the DigitalTwinPort framework under realistic disturbance conditions, such as network latency, signal interference, and hardware-in-the-loop testing. In addition, comprehensive quantitative benchmarking will be conducted to assess the performance and scalability of the proposed framework under realistic multi-node network scenarios. While the current demonstration focuses on Windows with Winsock, a minimal Linux demonstration is planned as part of future work. This will include testing the DigitalTwinPort framework in a ROS/ROS2 environment using POSIX sockets, with detailed setup instructions for Linux-based systems.

Furthermore, future work will involve extending the DigitalTwinPort framework to support key digital twin-specific services, including state synchronization, event logging, replay mechanisms, and versioning. These services will ensure more comprehensive support for digital twin requirements, such as time management, fidelity levels, and data provenance. A detailed exploration of how these services can be mapped to specific use cases, like Autonomous Surface Vehicles (ASVs), will also be part of the future work.

## Figures and Tables

**Figure 6:**
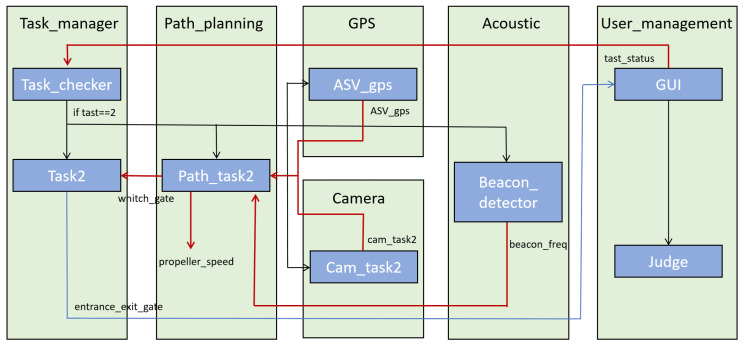
Node communication graph for Task 2 showing publisher-subscriber relationships between the seven core nodes and their associated topics.

**Table 1:** Comparison between DigitalTwinPort and Existing Middleware Frameworks.

Feature	DigitalTwinPort	ROS/DDS	MQTT/Other Bus
Naming and Discovery	Local, object-level; no global registry required	Global topic/service naming and discovery mechanisms	Broker-based or topic-level naming
QoS / Reliability	Lightweight handling integrated in object methods	DDS QoS profiles; ROS2 transport layer settings	Broker-configured QoS profiles
Schema / IDL Dependency	None (implicit data binding through class definition)	Requires predefined message/service definitions	Optional, depending on broker and payload format
Transport Layer	Extensible TCP/UDP sockets or custom protocols	DDS, TCPROS, Fast-RTPS	MQTT, WebSocket, HTTP, etc.
Configuration Overhead	Minimal (embedded in class initialization)	Requires external launch/configuration files	Requires broker setup and maintenance
Design Philosophy	Port-level abstraction mirroring hardware communication; biomimetic mapping	Message-centric middleware for distributed systems	Broker-centric message bus for IoT devices

## Data Availability

All data related to this paper will be made available upon request. The access to the data is subject to the data protection policy of Nanyang Technological University, Singapore.

## Abbreviations

The following abbreviations are used in this manuscript:

IoT	Internet of Things
ROS	Robot Operating System
SD	Smart Device
IPC	Inter-process Communication
ASV	Autonomous Surface Vehicle
SM	Software Module
